# Clinicopathologic significance of TRAP1 expression in colorectal cancer: a large scale study of human colorectal adenocarcinoma tissues

**DOI:** 10.1186/s13000-017-0598-3

**Published:** 2017-01-14

**Authors:** Min Gyoung Pak, Hyong Jong Koh, Mee Sook Roh

**Affiliations:** 1 Department of Pathology, Dong-A University College of Medicine, 26 Daesingongwon-ro, Seo-gu, Busan, 49201 South Korea; 2 Department of Parmacology, Dong-A University College of Medicine, Busan, South Korea

**Keywords:** TRAP1 (tumor necrosis factor receptor-associated protein 1), Colorectal cancer, Local invasion, Disease-specific survival

## Abstract

**Background:**

Colorectal cancer is the major cause of cancer mortality, despite development of therapeutic strategies. The novel marker tumor necrosis factor receptor-associated protein 1 (TRAP1) is a mitochondrial heat shock protein that has been related to drug resistance and protection from apoptosis in colorectal cancer. This study aims to delineate the clinicopathologic significance of TRAP1 expression in colorectal cancer.

**Methods:**

Seven-hundred and fourteen FFPE tissues were collected from colorectal cancer patients who underwent surgery from February 2002 to July 2011 at Dong-A University Medical Center, Busan, South Korea. We performed TRAP1 immunohistochemistry using tissue microarray, and divided into two groups, TRAP1 high expression group and low expression group. Statistical analysis was utilized to evaluate the association of TRAP1 with clinicopathologic characteristics and disease-specific survival of patients.

**Results:**

High TRAP1 expression was observed in 564 cases (79%) and low expression was 150 cases (21%). TRAP1 expression was significantly increased in colorectal cancer with advanced pathologic T-stage compared with that in early T-stage (*p* = 0.008). By univariate survival analysis, high TRAP1 expression was significantly associated with worse disease-specific survival (*p* = 0.01). But, TRAP1 expression was marginally associated with lymph node involvement and tumor differentiation (*p* = 0.085, *p* = 0.082, respectively). Multivariate analysis indicated that TRAP1 expression (hazard ratio, 1.947; 95% CI, 1.270 to 2.984; *p* = 0.002), and pathologic T stage (hazard ratio, 3.190; 95% CI, 1.275 to 7.983; *p* = 0.013) were independent prognostic factors for colorectal adenocarcinomas.

**Conclusions:**

Here, we found that overexpression of TRAP1 might contribute to tumor cell local invasion of colorectal cancer. The association between TRAP1 overexpression and worse disease-specific survival also suggested that TRAP1 protein expression might have oncogenic role. Consequently, our data demonstrated that TRAP1 expression was a good prognostic biomarker for depth of invasion and disease-specific survival in colorectal cancer.

## Background

Colorectal cancer is one of the most commonly diagnosed cancers along with breast, lung and bronchus cancers in men and women. The incidence of colorectal cancer rapidly declines with increased screening and removal of precancerous lesions by colonoscopy. Nevertheless, in 2016, colorectal cancer is still a major cause of cancer death in men and women [1]. There are no treatment options for advanced colorectal cancer patients after all approved standard therapies, but many patients maintain a good performance status and could be candidates for further targeted therapy [2].

Tumor necrosis factor receptor associated protein 1 (TRAP1) is a molecular chaperone of the 90 kDa heat shock protein (HSP90) family, and is mostly localized to the mitochondrial matrix. TRAP1 was first identified as a protein interacting with the intracellular domain of the type 1 TNF receptor. Further sequence analysis revealed that TRAP1 was identical to heat shock protein 75 (HSP75) [3]. Biologically, TRAP1 modulates the permeability transition pore of mitochondrial inner membrane, and protects the mitochondrial structure from excessive reactive oxygen species (ROS) induced cell death, such as in Parkinson’s disease and cancers [4, 5].

TRAP1 is undetectable or expressed at very low levels in normal tissue, but is up-regulated in various human malignancies, including ovarian carcinoma cell lines [6], colorectal carcinomas [7], high-grade prostatic intraepithelial neoplasia, Gleason grades three through five prostatic adenocarcinomas [8], and kidney cancer [9]. Recent studies show that TRAP1 is associated with positive lymph node metastasis [10], multi-drug resistance [7], and shorter median overall survival [11] in colorectal cancer. But there are still limited researches about relationship between TRAP1 expression and pathologic parameters in colorectal cancers.

In this study, we delineate clinicopathologic significance of TRAP1 using a large number of formalin-fixed paraffin-embedded (FFPE) specimens of colorectal cancers to investigate its potential as a biomarker, and analyze the prognostic significance of the TRAP1 status.

## Methods

### Patients and samples

Seven-hundred and fourteen FFPE tissues were collected from colorectal cancer patients, who underwent surgery from February 2002 to July 2011 at Dong-A University Medical Center, Busan, South Korea. Eligible samples were obtained from colorectal cancers without preoperative chemotherapy or radiotherapy history. Excluded were histologic types other than primary colorectal adenocarcinoma, and patients with co-existing malignancy.

Tissue microarrays (TMAs) were constructed from representative tissue from FFPE samples. All the pathologic slides were reviewed independently by two pathologists, and the histological type, location, tumor differentiation, depth of invasion, lymph node metastasis, and lymphovascular invasion were reevaluated. Clinical stage was re-determined according to the 7th edition of the American Joint Committee on Cancer TNM staging system [12]. Other clinicopathological data including age, sex, distant metastasis and survival data were obtained from medical records. Survival observation time was the time interval between surgery and the last contact (death due to colorectal carcinoma or last follow-up). Patients without recurrence or cancer progression were censored at the time of the last follow-up.

This study was approved by the Institutional Review Board (IRB) at Dong-A University Medical Center (DAMC IRB approval No.15-141). Written informed consent was obtained from all of the patients. All specimens were handled and made anonymous according to the ethical and legal standards.

### Immunohistochemistry

Sections from TMA blocks were transferred onto poly-L-lysine-coated glass slides. Sections from formalin-fixed paraffin-embedded blocks were 4 μm in thickness. Immunohistochemistry was performed using a Ventana BenchMark XT automated stainer (Ventana Medical Systems, Tucson, AZ, USA) according to the protocol with Endogenous Biotin Blocking Kits (Ventana Medical Systems). The fully automated processing of bar code-labeled slides included deparaffinization, and antigen retrieval techniques in EDTA buffer pH 8, and primary antibody incubation using the TRAP1 antibody (monoclonal mouse, Cat 612345, BD BioSciences, San Jose, CA, USA) at a titer of 1:100. Subsequent reactions with secondary antibody and visualization were achieved with DAB (UltraView Universal DAB Detection kit; Ventana Medical Systems). Formalin fixed, paraffin-embedded human endometrial tissue (endometrial glandular cells are positive) was used a positive control according to antibody manufacturer’s instruction. These sections were also counterstained with hematoxylin.

All TMA slides were evaluated independently by two pathologists using light microscopy in a double-blinded manner. Discordant cases were re-evaluated on a multi-headed microscope to achieve a consensus. TRAP1 expression was evaluated for each tissue sample by calculating a total immunohistochemistry score as the product of a proportion and intensity score [13]. The proportion score described the estimated fraction of positively stained tumor cells (0 = none; 1 = 1 ~ 25%; 2 = 26 ~ 50%; 3 = 51 ~ 75%; 4 = 76 ~ 100%). The intensity score represented the estimated staining intensity (0, negative; 1, weak; 2, moderate; 3, strong) of the samples. TRAP1 expression was evaluated for each tissue sample by calculating a final immunohistochemistry score as the product of the proportion score and the intensity score. The final immunohistochemistry score ranged from 0 to 12. The high-TRAP1 expression group was arbitrarily defined as a total score ≥ 6, while the low-TRAP1 expression group was considered as a total score < 6.

### Statistical analysis

The following clinicopathologic factors were examed: sex, age, tumor size, tumor differentiation, pathologic T, N, and M stage, pathologic TNM stage, lymphovascular invasion, and TRAP1 expression. Statistical analyses were conducted using the statistical software SPSS 18.0 for Windows (SPSS Inc., Chicago, IL, USA). The chi-square test was used to assess TRAP1 expression with respect to clinicopathologic parameters. The survival curves of the patients were determined using the Kaplan-Meier method, and the log-rank test. Cox’s proportional hazard model was used for statistical evaluations. Statistical significance was considered when probability(*p*)-value was less than 0.05.

## Results

### Clinicopathologic characteristics

This study included 714 colorectal adenocarcinoma patients representing 409 men and 305 women. The median age at diagnosis was 62 years (range, 22 to 87 years). The primary tumor locations included 456 (63.9%) in colon, and 258 (36.1%) in rectum. Histologically, 399 tumors (55.9%) were classified as well-differentiated adenocarcinoma, 270 (37.8%) as moderate-differentiated, 22 (3.1%) as poorly-differentiated, and 23 (3.2%) as mucinous carcinoma. Pathological T stage consisted of pTis for 5 tumors (0.7%), pT1 for 22 (3.1%), pT2 for 85 (11.9%), pT3 for 574 (80.4%), pT4a for 13 (1.8%), and pT4b for 15 (2.1%). Patients numbering at 164 (23%) had lymphovascular invasion, 306 (42.9%) had lymph node metastasis, and 43 (6%) had distant metastasis at the time of surgery, respectively. Details of clinicopathologic factors are indicated in Table 1.

**Table 1:** Relationship between TRAP1 expression and clinicopathologic factors

Clinicopathologic factors	Number	TRAP1 expression		*p* value
		Low^a^ (*n* = 150)	High^b^ (*n* = 564)	
Sex				
Male	409	90	319	0.107
Female	305	60	245	
Age (year)				
≤ 60	297	67	230	0.391
> 60	417	83	334	
Tumor Size (cm)				
≤ 5	342	64	278	0.149
> 5	372	86	286	
Tumor differentiation				
Well	399	78	321	0.085
Moderate	270	56	214	
Poorly	22	7	15	
Mucinous	23	9	14	
Pathologic T stage				
pTis	5	4	1	0.008
pT1	22	5	17	
pT2	85	12	73	
pT3	574	120	454	
pT4a	13	5	8	
pT4b	15	4	11	
Pathologic N stage				
pN0	408	84	324	0.101
pN1a	98	16	82	
pN1b	92	19	73	
pN1c	3	1	2	
pN2a	60	21	39	
pN2b	53	9	44	
Pathologic M stage				
pM0	671	139	532	0.280
pM1a	24	8	16	
pM1b	19	3	16	
pTNM stage				
0	6	4	2	0.082
I	90	15	75	
llA	293	59	234	
llB	2	1	1	
llC	4	0	4	
lllA	14	1	13	
lllB	211	46	165	
lllC	50	13	37	
IVA	25	8	17	
IVB	19	3	16	
Lymphovascular invasion				
No	550	111	439	0.321
Yes	164	39	125	

^a^total score < 6

^b^total score ≥ 6

### Association between TRAP1 expression and clinicopathologic factors

**Fig. 1:**
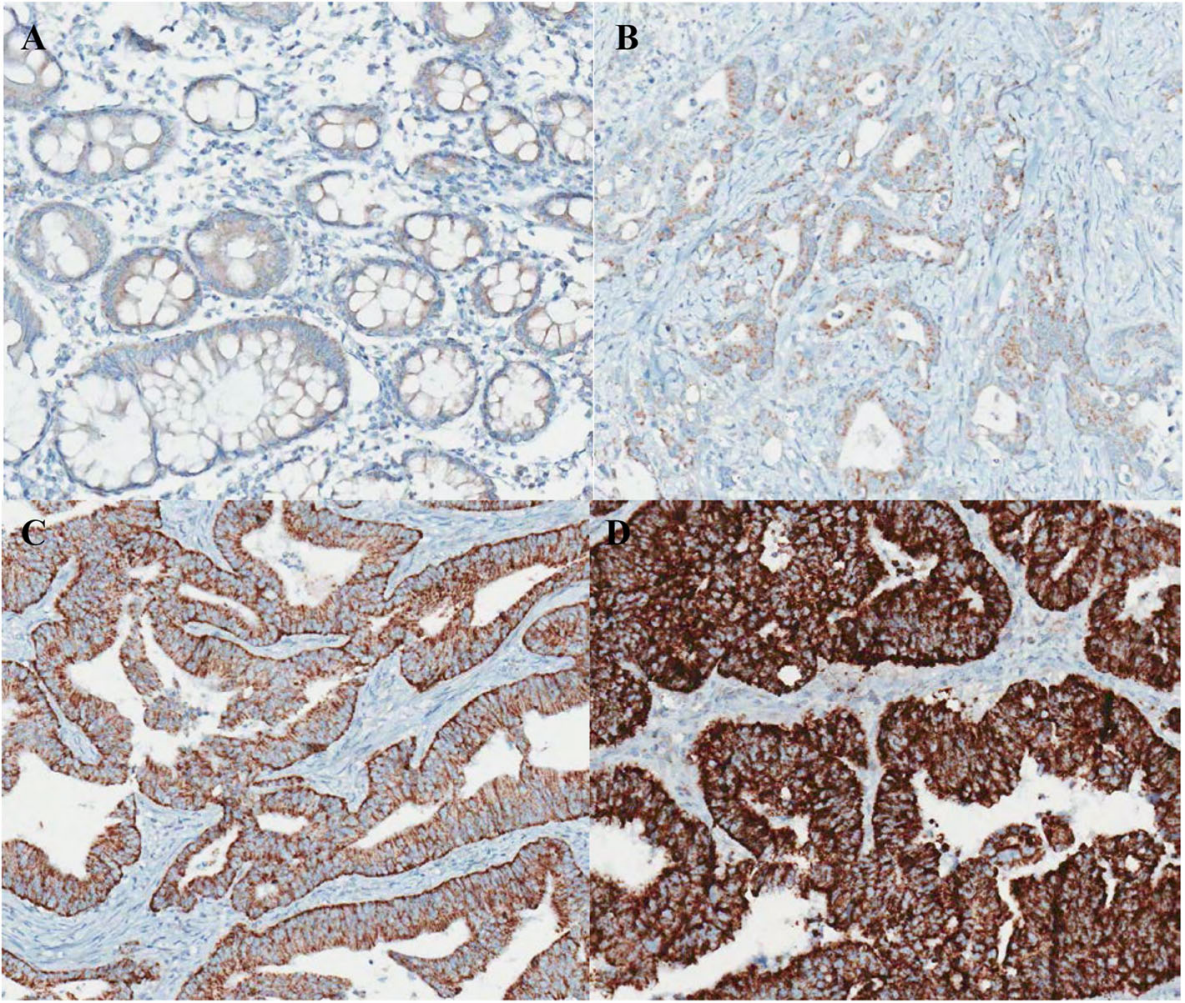
Intensity score of TRAP1 expression **a** Negative or very low expression of TRAP1 in normal colon **b-d** Various TRAP1 expression levels in colorectal adenocarcinomas **b** weak **c** intermediate **d** strong positive stainings

**Fig. 2:**
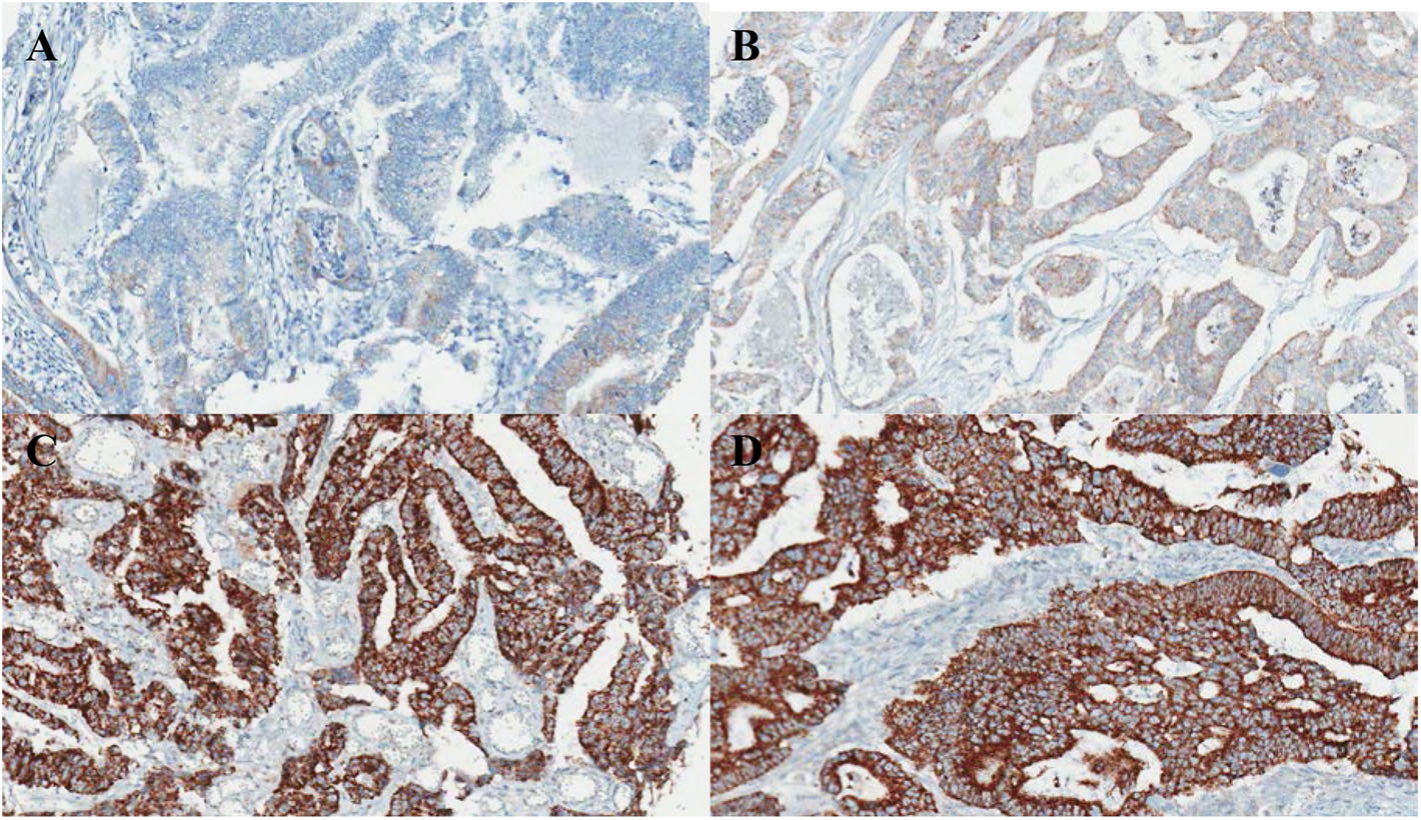
Results of TRAP1 expression **a-b** Low-TRAP1 expression **c d** High-TRAP1 expression

TMAs containing tissue from 714 colorectal adenocarcinomas were stained with anti-TRAP1 antibody, and the intensity of TRAP1 expression varied from weak to strong (Fig. 1b: weak, c: moderate, d: strong positive staining). As presented in Fig. 1, TRAP1 showed no staining or had very low expression level in normal colon tissue (Fig. 1a). In colorectal adenocarcinomas, high-TRAP1 expression was observed in 564 patients (79%) and 150 patients (21%) showed low-TRAP1 expression. Examples of high- and low-TRAP1 expressions are shown in Fig. 2. We evaluated the correlation between TRAP1 expression and various clinicopathologic factors to assess the diagnostic and prognostic significance of TRAP1. Chi-square test was used to assess TRAP1 expression with respect to clinicopathologic parameters. The results revealed that the pathologic T stage had a statistically positive correlation with TRAP1 expression (*p* = 0.008). In addition, worse tumor differentiation and increasing pathologic N stage were marginally associated with TRAP1 expression (*p* = 0.085, 0.082, respectively), but they were not statistically significant. Also, there were no significant associations between TRAP1 expression and other clinicopathologic factors of sex, age, tumor size, pathologic M, TNM stage, and lymphovascular invasion (*p* > 0.05, Table 1).

### Survival according to TRAP1 expression

**Fig. 3:**
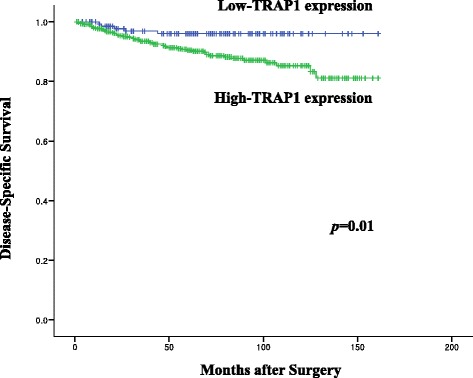
Kaplan-Meier survival analysis of disease-specific survival according to TRAP1 expression

The median follow-up period of the 714 patients was 64 months (range, 1 to 161 months). Among these 714 patients, 401 were followed-up for more than 60 months. For patients, 60 (8.4%) died from colorectal adenocarcinomas for a follow-up period. Among the dead, five of 150 were low-TRAP1 expressers and 55 of 564 belonged to the high-TRAP1 expression group. In addition, six patients died from pneumonia and one patient died from a car accident during the follow-up period. However, they were not included in the number of deaths for survival analysis. Kaplan-Meier survival analysis showed that patients with high-TRAP1 expression had significantly shorter disease-specific survival than those with low-TRAP1 expression (*p* = 0.01, Fig. 3). Further multivariate analysis was performed using Cox regression model to determine independent prognostic factors for colorectal adenocarcinomas. Relevant clinicopathologic factors including sex, age, tumor size, tumor differentiation, pathologic T, N, and M stage, pathologic TNM stage, lymphovascular invasion, and TRAP1 expression were analyzed in the Cox univariate model. Only those factors with a *p* value <0.01, pathologic T stage and TRAP1 expression, were included in the multivariate model. Cox proportional hazard regression analysis indicated that TRAP1 expression (hazard ratio, 1.947; 95% CI, 1.270 to 2.984; *p* = 0.002), and pathologic T stage (hazard ratio, 3.190; 95% CI, 1.275 to 7.983; *p* = 0.013) were independent prognostic factors for colorectal adenocarcinomas (Table 2).

**Table 2:** Cox proportional hazard regression analysis in colorectal adenocarcinoma

Variables	Hazard ratio	95% Confidence interval	*p* value
TRAP1 expression	1.947	1.270–2.984	0.002
Pathologic T stage	3.190	1.275–7.983	0.013

## Discussion

It is generally accepted that human malignant tumors develop by genetic alterations and are also composed of heterogeneous population of cells. Previous studies have shown that colorectal cancer is also a genetically heterogeneous and complicated disease [14]. Numerous therapeutic regimens including target therapies for colorectal carcinomas have been proposed, but with all the currently approved standard therapies, the disease is still progressive for the majority of patients.

Recently, several investigations have shown that TRAP1 is a significant factor related to metastasis and prognosis in colorectal cancers. Gao et al. found that TRAP1 was significantly up-regulated in primary colorectal cancers with lymph node metastasis compared with lymph node negative ones [10]. Costantino et al. identified that TRAP1 overexpression lead to 5-fluorouracil-, oxaliplatin- and irinotecan-resistant phenotypes in neoplastic cells [7]. Han et al. demonstrated that the median overall survival was significantly increased in patients negative for TRAP1 than those positive for TRAP1 [11]. Despite these findings, investigations at a large scale from human colorectal tissues have been limited to evaluate the relationships between TRAP1 expression and colorectal cancers with various clinicopathologic parameters.

This study showed that the pathologic T stage had a statistically positive correlation with TRAP1 expression. In the view of this finding, it seems reasonable to regard that TRAP1 expression contributes to local cancer invasion. As far as we know, this is the first study to evaluate the relationship between TRAP1 expression and local cancer invasion. From these findings, we suggest that TRAP1 enables tumor cells to invade stromal tissue by epithelial-mesenchymal transition (EMT). As TRAP1’s full name, tumor necrosis factor receptor-associated protein 1 implies [9], TNF-α promotes tumor invasion via induction of matrix metalloproteinases, and finally modulates EMT in a model of colorectal cancer [15]. In addition, TRAP1 inhibits the enzymatic activity of succinate dehydrogenase (SDH), and SDH inhibition leads to succinate-dependent hypoxia-inducible factor 1-alpha (HIF1-a) stabilization [16]. HIF1 stabilization contributes to neoplastic processes by EMT [17], and EMT plays a critical role in migration of tumor cells from the primary site into stromal tissue [18]. The precise pathologic mechanisms for TRAP1 in promoting cancer invasion are still not fully understood and many questions remain to be answered. But TRAP1 seems to be one of the critical players in biologic processes of tumor invasion in colorectal cancer.

In this study, an increasing pathologic N stage was marginally associated with TRAP1 expression. In addition, it is known that TRAP1 expression is significantly associated with lymph node metastasis in colorectal cancers [10], prostatic cancers [8], and esophageal squamous cell carcinomas [19]. These findings are in disagreement with those of Kubota et al. who recently reported that TRAP1 regulates cell adhesion by modulation of N-cadherin expression in a neuronal cell line [20]. The aforementioned study showed that the cell scattering phenotype in the TRAP1 knockdown cells could be mainly attributed to impaired expression of cell adhesion molecules including N-cadherin, which is regulated by TRAP1 transcription. In our opinion, the two different results may be due to the different microenvironments in the models studied. Tumor hypoxia can be found in almost every solid tumor, and gene expression along with associated biological function can alter according to circumstances such as stressful tumor microenvironment [21].

In future sudies, expressions of TRAP1 in different parts of the same tumor, especially in the front of invasion where it has contact with the microenvironment, need to be determined. Such study will help us elucidate the relationship between TRAP1 expression and the tumor microenvironment.

In this study, tumor differentiation showed a marginal positive correlation with TRAP1 expression. This finding is similar with that of Li et al. who reported a strong correlation with positive TRAP1 staining and the World Health Organization (WHO) grade [22]. In the present study, patients with high-TRAP1 expression showed significantly shorter disease-specific survival than those with low-TRAP1 expression. This finding is in accord with an earlier one on colorectal cancer cases treated with oxaliplatin/5-fluorouracil [11]. Significant positive correlations between high-TRAP1 expression and poor prognosis were observed not only in this study but also in other studies. TRAP1 expression levels were also increased in cisplatin-resistant ovarian carcinoma cell lines [6], colorectal carcinoma cells resistant to 5-fluorouracil [7], oxaliplatin and irinotecan [7], and breast carcinoma cells resistant to paclitaxel [23]. In addition, high -TRAP1 expression level was also associated with increased risk of disease recurrence in non-small cell lung cancer [24]. It is reasonable that TRAP1 contributes to tumor progression based on these results. In a stressful environment, permeability transition pore of mitochondrial inner membrane is opened by accumulated ROS, and consequently cells undergo apoptosis [4]. TRAP1 protects tumor cells from ROS-dependent permeability transition pore opening through deregulated mitochondrion-restricted kinase signaling [17]. This may be the key mechanism of TRAP1-related tumor progression.

Our multivariate analysis showed that TRAP1 expression was useful for predicting the clinical outcome of colorectal adenocarcinomas. Therefore, TRAP1 expression can be used as an additional novel prognostic marker of colorectal adenocarcinomas.

We revealed that up-regulation of TRAP1 might plays a major role in biological functions. It particularly influenced colorectal cancer invasion and disease-specific survival. Until now, only a limited number of studies have delineated the pathophysiologic functions of TRAP1 and relationships between TRAP1 and clinicopathologic factors including histology in human colorectal cancer tissue. Our study provides new and important information towards understanding the pathogenesis of colorectal adenocarcinomas. Further investigations are required with respect to discordant views and precise pathophysiologic molecular mechanisms.

## Conclusion

In conclusion, we clarified the relationship between TRAP1 expression and clinicopathologic factors, and evaluated diagnostic and prognostic significance of TRAP1 as a potential biomarker, using a large number of human colorectal adenocarcinoma FFPE samples.

To the best of our knowledge, this is the first large scale study revealing the relationships between TRAP1 expression in surgically resected specimens with various clinicopathologic factors and long term follow-up records. Our study showed that TRAP1 was associated with local invasion in colorectal adenocarcinomas. Our results firmly indicated that TRAP1 was as a novel independent prognostic factor for colorectal adenocarcinomas. It has been recently demonstrated that TRAP1 is responsible for a multi-drug resistant phenotype in human colorectal carcinoma cells [7]. This information confers that TRAP1 expression is not only a prognostic factor but also a predictive factor for colorectal cancer. Further studies on TRAP1 expression with clinical trial might contribute to building a new era of personalized medicine in treatment for colorectal cancer.

## Abbreviations

CI: Confidence interval
EMT: Epithelial-mesenchymal transition
FFPE: Formalin-fixed paraffin-embedded
HIF-a: Hypoxia-inducible factor 1-alpha
HSP75: Heat shock protein 75
Hsp90: Heat shock protein 90
ROS: Reactive oxygen species
SDH: Succinate dehydrogenase
TMAs: Tissue microarrays
TRAP1: Tumor necrosis factor receptor associated protein 1
